# XRF calibration with low-cost samples and implementation for quantification of inorganic elements in lipsticks

**DOI:** 10.1016/j.mex.2024.102704

**Published:** 2024-04-07

**Authors:** M Pilakouta, M Trapali, N Kallithrakas-Kontos

**Affiliations:** a University of West Attica, Department of Biomedical Sciences, Campus 1, Ag. Spyridonos 28, Egaleo 12243 Athens, Greece; b Analytical and Environmental Chemistry Laboratory, School of Mineral Resources Engineering, Technical University of Crete, University Campus, GR 73100 Chania, Greece

**Keywords:** XRF calibration, Educational activity, Thin sample, Cosmetics, Lipsticks, Preparation of thin standards for calibration

## Abstract

In this paper, the preparation of low-cost samples, for the calibration of an energy–dispersive X-ray fluorescence system is presented. The entire procedure is proposed as an undergraduate or postgraduate student laboratory activity, which aims to familiarize students with the procedure of calibration of a spectroscopy-based analytical method through the XRF technique. Furthermore, the use of XRF for the determination of elemental concentrations in samples related to students’ interests, was attempted.

## Graphical abstract

**Figure:**
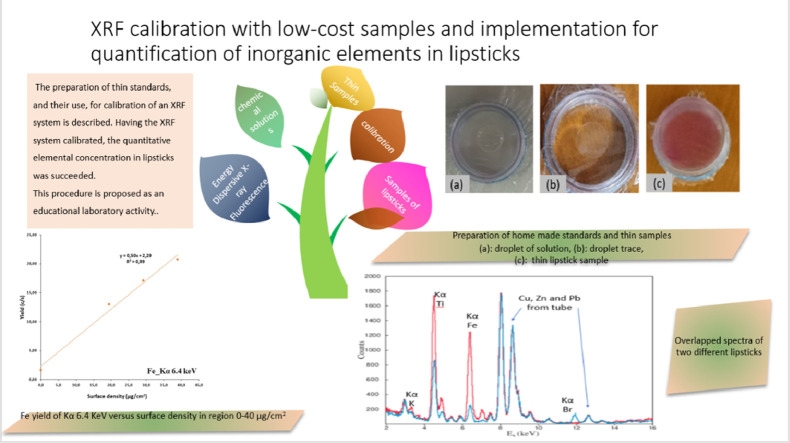

Specifications table

**Table:** 

Subject area:	Chemistry
More specific subject area:	A spectroscopy-based analytical method
Name of your protocol:	XRF calibration with homemade standards
Reagents/tools:	Solutions from several compounds of the elements of interest (99% initial chemical compound purity) were used to prepare monoelemental homemade standards.
	EDXRF portable system
Experimental design:	Preparation of standard solutions
	Preparation of home made thin standards on a membrane.
	Calibration of XRF spectrometer using home made standards.
	Preparation of lipsticks samples
	Determination of elemental concentration in lipsticks
Trial registration:	N.A
Ethics:	N.A
Value of the Protocol:	▪ the preparation of low - cost home made standards, for the calibration of an energy–dispersive X-ray fluorescence system
	▪ the use of XRF for the determination of elemental concentrations in samples related to students’ interests
	▪ familiarization of students with the procedure of calibration of a spectroscopy-based analytical method through the XRF technique

## Background

Energy Dispersive X-ray Fluorescence (XRF) is an atomic spectrometric technique used in a wide range of applications. It has the advantage of simultaneous analysis of a wide range of elements, from atomic number 11 and above (depending on the system) and from percentages up to ppm (mg/kg) concentrations. It is fast, non-destructive in many cases and needs almost no sample preparation [1], [2], [3]. XRF technique has been used as an educational tool for more than 30 years and is often incorporated into educational activities in several areas, chemistry, physics, geology, archaeometry, and environmental sciences [4], [5], [6], [7], [8], [9], [10], [11], [12], [13].

The element identification from the energy spectrum is relatively easy, especially for the intense peaks and is ideal for a quick classroom demonstration. On the contrary, the quantitative analysis is not straightforward. To derive the elemental concentrations from the peak intensities, the x-ray attenuation factors in the sample, the enhancement and interferences among elements etc. must be considered [2,9].

The quantification is much easier in case of examining thin samples [14], i.e., samples with negligible X-ray self-absorption, since the only needed is to calibrate the XRF system using thin reference standards, commercial or homemade.

Our students in the Department of Biomedical Sciences of UNIWA (DBS), have a special interest in the elemental concentrations of pharmaceutical /chemical as well as of cosmetic products (Medical laboratories Science, Aesthetics and Cosmetic Science divisions) from the point of view of toxicology and product safety control.

The quantification of inorganic impurities within a product is one of the most common analytical tests. Toxic heavy metals, such as arsenic (As), cadmium (Cd), mercury (Hg), and lead (Pb) as well as other metals, such as iron (Fe), chromium (Cr), nickel (Ni) and zinc (Zn), are of interest due to health risks. Furthermore, it is interesting from the didactic point of view, to examine and discuss the origin and the purpose of the existence of the minerals in samples of everyday life.

In order to include the XRF technique in our students’ curriculum, some experimental activities including the calibration procedure, have been designed and tested.

In this paper, the preparation of thin Homemade standards (Homas) for the XRF calibration using solutions of various chemical compounds is presented. The calibration of the XRF system is explained/and finally, results of quantitative analysis of trace elements in some lipsticks, that can be treated as thin samples, are presented and discussed.

The whole procedure can be used in an undergraduate or postgraduate student laboratory. The educational activity aims to familiarize students (in the biomedical sciences field) with XRF methodology and the procedure of calibration, which is similar and for other spectroscopy-based analytical methods. Furthermore, the advantages and the possibilities of portable XRF as a qualitative and quantitative tool for elemental characterization of a great variety of samples may highlighted as well as the use of this technique for the quality control of chemical/pharmaceutical/cosmetic products.

In this interdisciplinary experimental activity instructors have the chance to discuss various subjects:

• the atomic structure, the x-rays, the excitation procedure and the origin of fluorescence,
• the instrumentation used in x-ray spectrometry,
• the preparation of chemical solutions and how this basic and routine, procedure may be used for the preparation of calibration standards (Homas),
• the importance of calibration of an analytical method,
• XRF sensitivity calibration, detection limits, precision and accuracy,
• the determination of elemental concentration in samples of chemical/biochemical interest including lipsticks.

In DBS, this activity has been already tested in an undergraduate special course in Biophysics and Toxicology. It can be also fit in courses in Special Biochemistry, Principles of Instrumental Analysis, as well as, in General and Inorganic Chemistry. In the postgraduate studies may fit to the course Modern Analytical Methods.

The experimental activity, (preparing solutions and Homas, examination of Homas, final XRF sensitivity calibration, sample analysis, spectra analysis time), including one-hour theoretical background, can be delivered in three laboratory sessions, 3 h each session. Additional 3 h for students to elaborate their report are needed.

A brief description of each step, as well as some results from the implementation of this activity, are presented in the following sections.

### X-ray fluorescence physical principles_ thin sample approximation

In brief, the physical principle of XRF, is based on atomic physics and the interaction of ionizing radiation with matter. When a material absorbs radiation of sufficient energy, some of the atoms are excited or ionized. After a short time, ∼10^–9^ s, atomic reorganization takes place and characteristic atomic radiation is emitted. The spectrum generated in the energy-dispersive XRF contains characteristic X-ray lines, scattering peaks, and a continuum background (bremsstrahlung radiation). Each X-ray line is denoted by the letter of the vacant electron shell (K, L, M) followed by Greek letters (a, b, etc.) indicating the shell from which the electron was donated. Thus when a vacancy in the K shell is filled by an electron coming from an L level, the transition is accompanied by the emission of an X-ray line known as the Kα line. On the other hand, if the atom contains sufficient electrons, the K shell vacancy might be filled by an electron coming from an M level that is accompanied by the emission of the Kβ line. The vacancies that remain in the L or M shell may also give rise to emission if the electron vacancies are filled by electrons falling from further orbits (La, Lβ, etc.).

This characteristic X-ray radiation can be used to identify the chemical elements present in the sample. As mentioned, element identification from the energy spectrum, in case of intense peaks of interest, is relatively easy, but an accurate quantitative analysis is much more difficult.

One of the methods used for obtaining quantitate results is the calibration of XRF using standards with known chemical composition. Such standards are generally very expensive and not always affordable for an educational lab.

An interesting approximation that is ideal for educational purposes, is the one of thin samples. In this case, XRF should be calibrated with thin standards, and the quantitative evaluation of elemental concentrations is extracted from a simple relationship between the fluorescence intensity of the characteristic Kα or Lα (for heavier elements) Χ-ray lines and the surface density of an element in the sample, according to the equation [15]:

$$c_{i}=\frac{I_{i}}{s_{Fi}}$$

where Ii is the characteristic X-ray net intensity in counts per second (cps), Ci concentration in (µg·cm^−2^) and S_Fi_ the elemental sensitivity factor (cps·µg^−1^· cm^2^), of the analyzed element i.

The concentration of an element in a sample can be expressed in parts per million (ppm) by dividing the element's concentration (Ci) in micrograms per square centimeter (μg.cm^-2^) by the sample's surface density in grams per square centimeter (g·cm^−2^). The preparation cost of thin Homas is very low and can be executed in a chemistry lab using appropriate chemical solutions.

## Description of protocol

### Samples

#### Preparation of thin homas

Solutions from several compounds of the elements of interest (99% initial chemical compound purity) were prepared in the chemistry lab. The compounds that were tested are listed in Table 1a, Table 1b.

**Table 1a:** Compounds used successfully to prepare calibration solutions.

Compound	Element	Mass (g)	Kα (keV)	
KI, KBr	K	39	3.3	
FeCl_3_	Fe	56	6.4	
Cu(NO_3_)_2_ 3H_2_O	Cu	64	8.0	
Ga_2_O_3_	Ga	70	9.2	
KBr	Br	80	11.9	
Sb_2_O_3_	Sb	122	26.4	
KI	I	127	28.6	
			Lα (keV)	
Pb(NO_3_)_2_	Pb	207	10.6

**Table 1b:** Compounds not successfully used for Homas preparation.

Compound	Element	Mass (g)	Kα (keV)
TiO_2_	Ti	48	4,5
NiCl_2_·6H_2_O	Ni	59	7,5
ZnCl_2_	Zn	65	8,6
MoO_3_	Mo	96	17,4
SnSO_4_	Sn	119	25,3
			Lα (KeV)
BaCO_3_	Ba	137	4,46
La_2_O_3_	La	139	4,65

Table 1a, contains the compounds that were used successfully, for standard solution preparation from which the Homas corresponding to the element X (second column) was made.

In Table 1b the compounds not successfully used for Homas preparation are listed.

The selected elements cover a region of Kα energies from 3.3 to 29 KeV corresponding to elements potassium (K) to iodine (I). Furthermore, heavier elements were also prepared for using Lα lines.

Water solutions of the compounds listed in Table 1 (K, Fe, Cu, Br, Sn, I and Pb) in several concentrations from 0.5 to 5 × 10^−3^ mol/L were prepared. Citric acid solutions (0.17 M) of compounds of Ga, Sb, La, Mo and Ba were prepared and further diluted in water to prepare the final concentrations from 0.5 to 5 × 10^−3^ mol/L. Each standard solution was used for the production of Homas of a specific element (second column of Table 1 a,b), except of the case of KBr and KI. In last column of Table 1a, Table 1b, the corresponding Kα or Lα X-ray line for each element is shown.

**Fig. 1:**
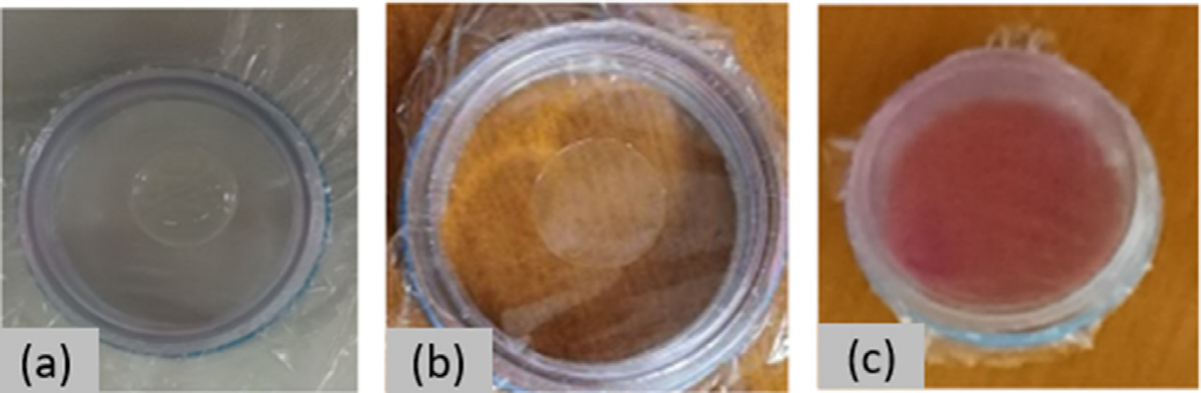
(a): droplet of solution, (b): droplet trace, (c): lipstick sample.

The Homas samples, “Fig. 1(a) and (b)”, are characterized as appropriate for using them as standard, only if they meet two prerequisites. The first is the diameter of the droplet trace (after drying physically at least 24 h) “Fig. 1(b)”, to be larger than the beam spot, which is ∼ 0.4 cm diameter when using a 2 mm collimator in X-ray tube, and secondly to be homogeneous.

For achieving an appropriate diameter of the droplet trace several Homas, with different volumes of each solution were prepared and tested. Volumes from 40 to 700 µL of the calibration solutions were placed onto thin-film sample support (Prolene 4.0 µm), and attached to a sample holder (double open-ended sample cups) using a micropipette. It was found that in a volume range of 200 – 500 µL, the “diameter” of the trace left after drying was between 0.7 to 1.5 cm, (corresponding to areas in the range of 0.38 to 2.5 cm^2^) which is quite larger than the beam spot (∼ 0.4 cm).

For selecting the most homogeneous Homas, for each element and each concentration, at least three Homas were prepared. The homogeneity of the prepared (dry) Homas was examined firstly by glance. If were looking homogeneous, then were checked by XRF, irradiating each one in two or three random positions. The accepted Homas were the Homas in which the yield differentiations (under the same experimental conditions) were in the range of the statistical uncertainty and no more than 10%. The adopted yield was the average of these measurements.

It should be mentioned that the attempt to prepare Homas for Ti, Ni, Zn, Mo, Sn, Ba and La was not successful due to homogeneity issues.

The Homas surface density was estimated from the mass of the element of interest in the solution, divided by the area of the “trace of the droplet”. The mass of each element was calculated from the solution concentration and the volume that had been used in each case. The accuracy of the thin Homas is mainly determined by their homogeneity and the accuracy of their surface density and is estimated of the order of 15%.

#### Lipsticks samples

Samples of lipsticks of different commercial brands collected by students, were analyzed without any preparation, applying a thin layer of each sample over the same thin membrane used for the Homas, supported by an XRF cup, “Fig. 1(c)”, leave them to dry, and placed over XRF system. The examined samples had concentrations less than 8 mg·cm^−2^ and were treated as “thin targets”.

### Experimental set up

**Fig. 2:**
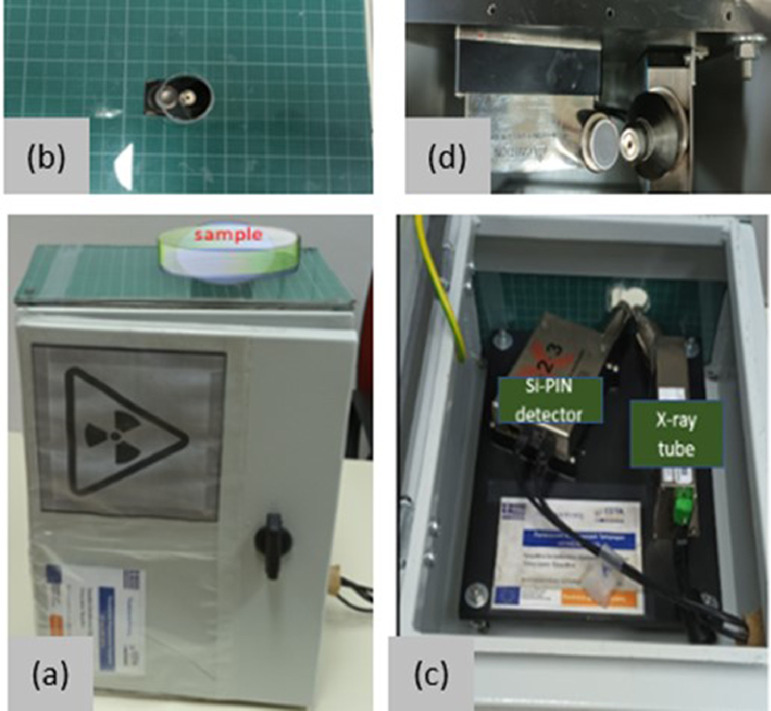
Experimental setup. (a) portable XRF system, (b) upper view of the case, (c) inside configuration and layout, (d) inside upper view.

The accepted Homas were examined with our XRF system (Amptek Experimenter's XRF Kit), as shown in “Fig. 2”, under the same geometry, (standard 45° XRF geometry, for both the angle of incidence and detection) and experimental conditions. The distance between the sample and the experimental setup was 1 cm, a 25 µm Al filter and a 2 mm collimator were used in the X-ray tube.

For the purpose of this educational activity, the use of only one irradiation condition was selected for sake of easiness and time restrictions. The irradiation at 40 keV and 40 µΑ was selected as a compromise experimental condition for having adequate excitation in a wide range of elements and concentrations. Under these experimental conditions the measurement time was ranging from 100 to 1000s.

### Calibration and quantification procedure

The spectra can be analyzed using any appropriate software, even the Amptek Inc. DppMCA Windows application which although, is primarily display and acquisition software can perform some basic analysis. Here the SPECTRW software was used [16] and analysis was performed by the instructors in our case.

To extract the elemental sensitivity calibration factor (S_Fi_) for element i, in cps.µg^−1^.cm^2^, at least three Homas with different concentrations were used. The S_Fi_ was extracted from the linear regression of the measurements of the three Homas and the blank measurement (empty membrane irradiation).

### Students’ engagement

#### First laboratory session

For the first laboratory session,14 students were separated into 7 groups and in each group was assigned to prepare solutions for two of the elements, of Table 1a, in three given concentrations for each element. The next step was the preparation of some Homas, as mentioned in 2.2.1 subsection and leaving them to dry. For each element and concentration, they prepared two Homas.

#### Second laboratory session – demonstration of the calibration procedure

**Fig. 3:**
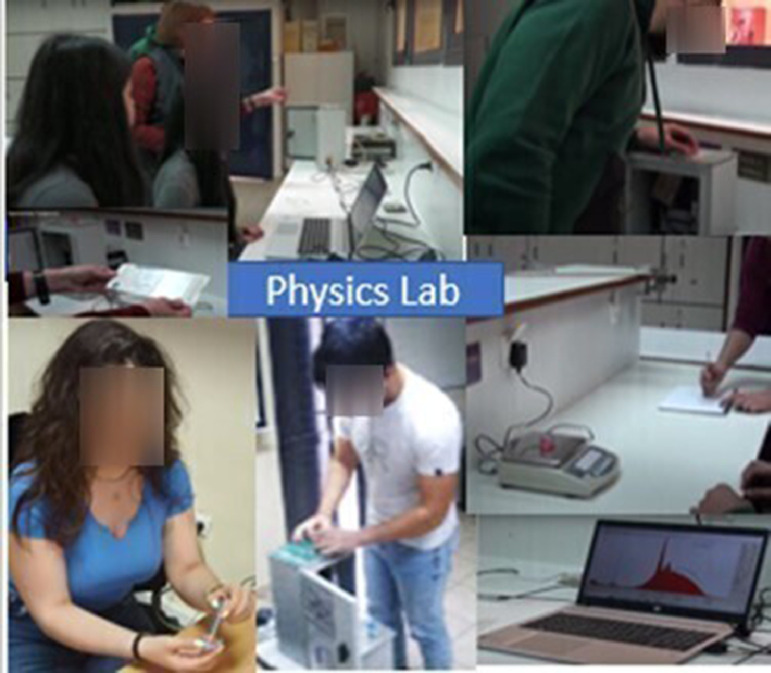
Students preparing and analyzing samples in the physics lab.

In the second lab session, after checking homogeneity by glance, they select the most homogeneous-looking Homas with the largest area of the trace of the drop. Students, with the help of educators, continue with more precise checks, with XRF, taking measurements in three different points of each Homas and selecting the most appropriate Homas to be used as calibration standards. Then they proceed to the sensitivity calibration of the XRF system, finding the S_Fi_ for each element. Finally, they prepare their own samples (lipstics, cremes, toothpastes) and leave them to dry physically “Fig. 3”.

#### Third laboratory session

In the last session with the help of educators they measure and analyze their own samples lipsticks, or other products (toothpastes, face crème, makeup) that can be treated as thin samples “Fig. 3”.

#### Protection from potential hazards

For the first session safety latex gloves should be worn when preparing solutions and samples, since some of them are toxic. Also, before finishing the laboratory session, students should dispose the waste in the proper disposal vessel.

XRF measurements (second and third sessions) are realized under the strict supervision of instructors. Students are informed beforehand that the experimental setup produces ionizing radiation and they should keep an appropriate safety distance during the irradiation of samples. The safety distance depends on the XRF system shielding. In our system, the safety distance is about 0.5 m.

### Calibration procedure

As previously mentioned, for all the elements, several Homas in a range of low concentrations were prepared to check the linearity of yield (cps) with surface density (µg/cm^2^). The most homogenous samples were measured and the adopted S_F_ for each element was extracted from the graphs with the best linearity.

**Fig. 4:**
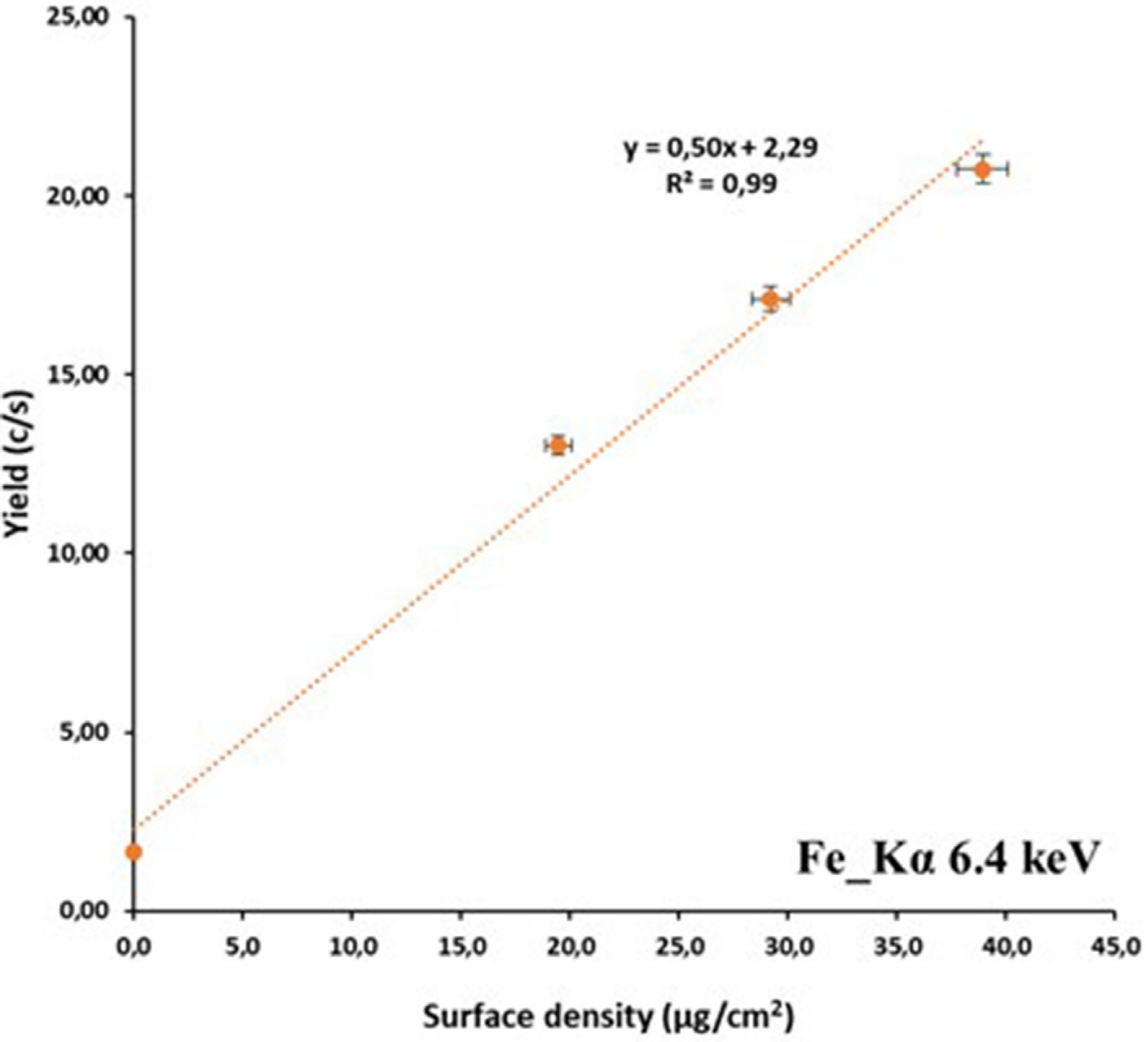
Fe yield of Kα 6.4 KeV versus surface density in region 0–40 µg/cm^2^.

**Fig. 5:**
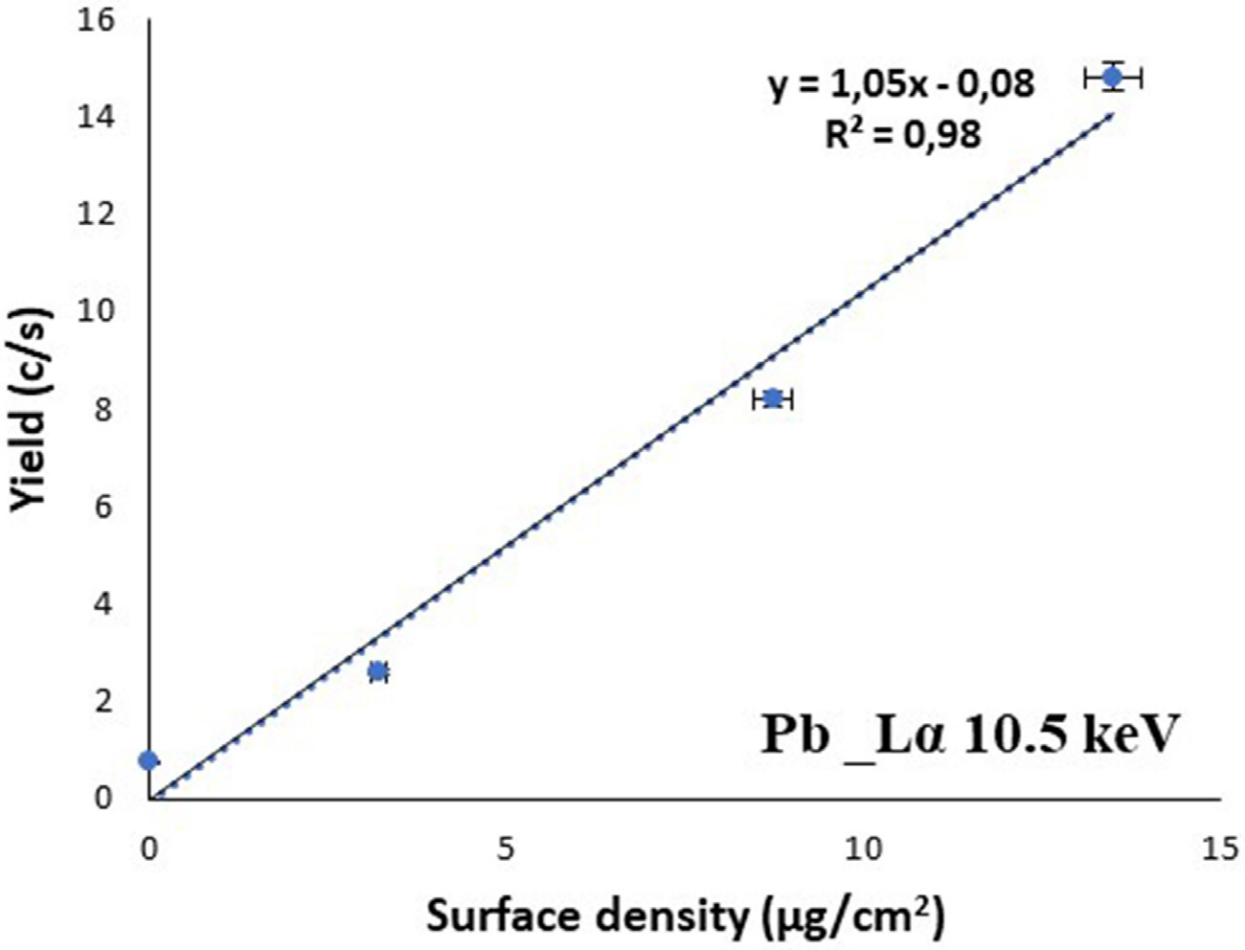
Pb yield of Lα versus surface density in the region of 2 to 16 µg/cm^2^.

Two graphs showing the linearity between yield and surface density for the prepared Homas, one for Kα line (6.4 keV) of iron (Fe) (“Fig. 4”) and another from Lα line (10.5 keV) of lead (Pb), (“Fig. 5”), are presented.

From the Fe graph, “Fig. 4”, it is obvious that in the region 0–40 µg/cm^2^ the linearity is excellent (R^2^=0.99) and a sensitivity factor 0.5 cps.mg^−1^cm^2^ for 6.4 keV was extracted.

In the case of Pb, 10.5 keV (La line), the adopted SF, was extracted from samples having surface density in the region 2 to16 µg/cm^2^ as it is shown in “Fig. 5”.

In all cases, either using yield from Kα (K, Fe, Cu, Ga, Br, I, Sb) or Lα (Pd), the linearity between yield and surface density was satisfactory. It should be mentioned that the S_F_ of potassium was estimated as the mean value deduced from KBr and KI Homas.

**Fig. 6:**
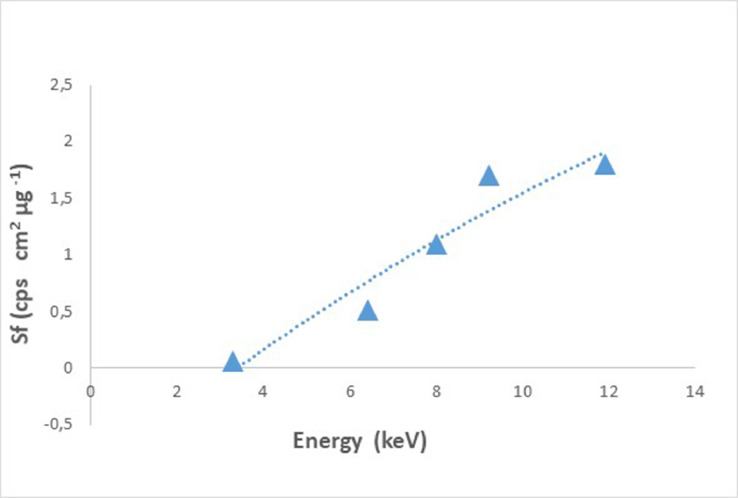
Sensitivity calibration curve extracted from (Kα lines) Homas.

In “Fig. 6”, the sensitivity curve of the XRF system extracted using Homas is presented (energy region 3–14 keV) and the fit of these data with a second-order polynomial function.

Using the sensitivity calibration curve, the S_F_ factors of other elements can be extracted (Ti, Rb etc.).

For some of the Homas (Cu, Ga, Br, Sb and Pb) a commercial standard (micromatter) was available. In Table 2 the S_F_ extracted from the successfully prepared Homas is compared with the S_F_ from available commercial standards. The differences are within 6 - 20%.

**Table 2:** Sensitivity factor comparison.

Εlement	Κα (keV)	S_F_ from Homas	S_F_ from Micromater stantards	Difference%
Cu	8.0	1.1	1.37	20
Ga	9.2	1.7	1.80	6
Br	11.9	1.8	2.04	12
Sb	26.4	0.5	0.42	19
	Lα (keV)			
Pb	10.6	1.05	1.26	17

Table 3, contains the S_F_ extracted directly from our Homas as well as S_F_ for some other elements (in the energy region 3–14 keV) that were estimated from the function that fits the sensitivity curve (Fig. 6).

**Table 3:** S_F_* extracted from Homas. Estimated S_F_ for some elements in the energy region (3 to 14 keV).

Element	Kα (keV)	Sensitivity factors
K	3.3	0.06 *
Ca	3.6	0.12
Ti	4.5	0.25
Fe	6.4	0.5 *
Cu	8.0	1.1 *
Zn	8.6	1.3
Ga	9.2	1.7 *
Br	11.9	1.8 *
Rb	13.4	2.2
Sr	14.2	2.1
Zr	15.8	1.7
Sb	26.4	0.5 *
I	28.6	0.18 *
	Lα (KeV)	
Pb	10.6	1.05 *

⁎ S_F_ extracted form Homas. All the other were estimated from sensitivity curve.

## Protocol validation

### Identifying elements in some lipsticks

Each student brings his own samples, mainly, lipsticks, lip gloss, body creams etc. Here we present results from 10 lipsticks of different commercial brands.

The excitation was performed at 40 kV and 40 µΑ current for 1000s analysis time.

The detected elements and their concentrations are listed in Table 4. The range of concentration (ppm) resulted from these 10 samples were for potassium K (3 samples) 8400 - 9600, for Ti (all samples) 8 × 10^3^- 100 *10^3^ for Fe (all samples) 620 – 37×10^3^, for Br (5 samples) 480 – 2250. Rb was detected only in one of the samples.

**Table 4:** Elemental concentration in the lipsticks.

Sample	Concentration of elements in ppm				
	K	Ti	Fe	Br	Rb
S1	ND	8000	2300	ND	ND
S2	ND	20,400	620	570	ND
S3	9000	27,000	23,000	ND	20
S4	8400	24,500	1150	530	ND
S5	9700	35,000	2350	480	ND
S6	ND	7300	37,000	ND	ND
S7	ND	100,000	35,000	ND	ND
S8	ND	5400	1900	ND	ND
S9	ND	35,200	26,400	2250	ND
S10	ND	74,000	35,500	2000	ND

There is a wide range of concentrations in elements, (especially Ti and Fe) among the samples that is may related to the color or color tone of each sample [15]. Αll the samples were in shades of red, purple, pink except for S7 which was beige. The estimated combined uncertainties (due to standards (15%), analysis (5%), and homogeneity of the lipstick samples (10%)) are of the order of 20%.

**Fig. 7:**
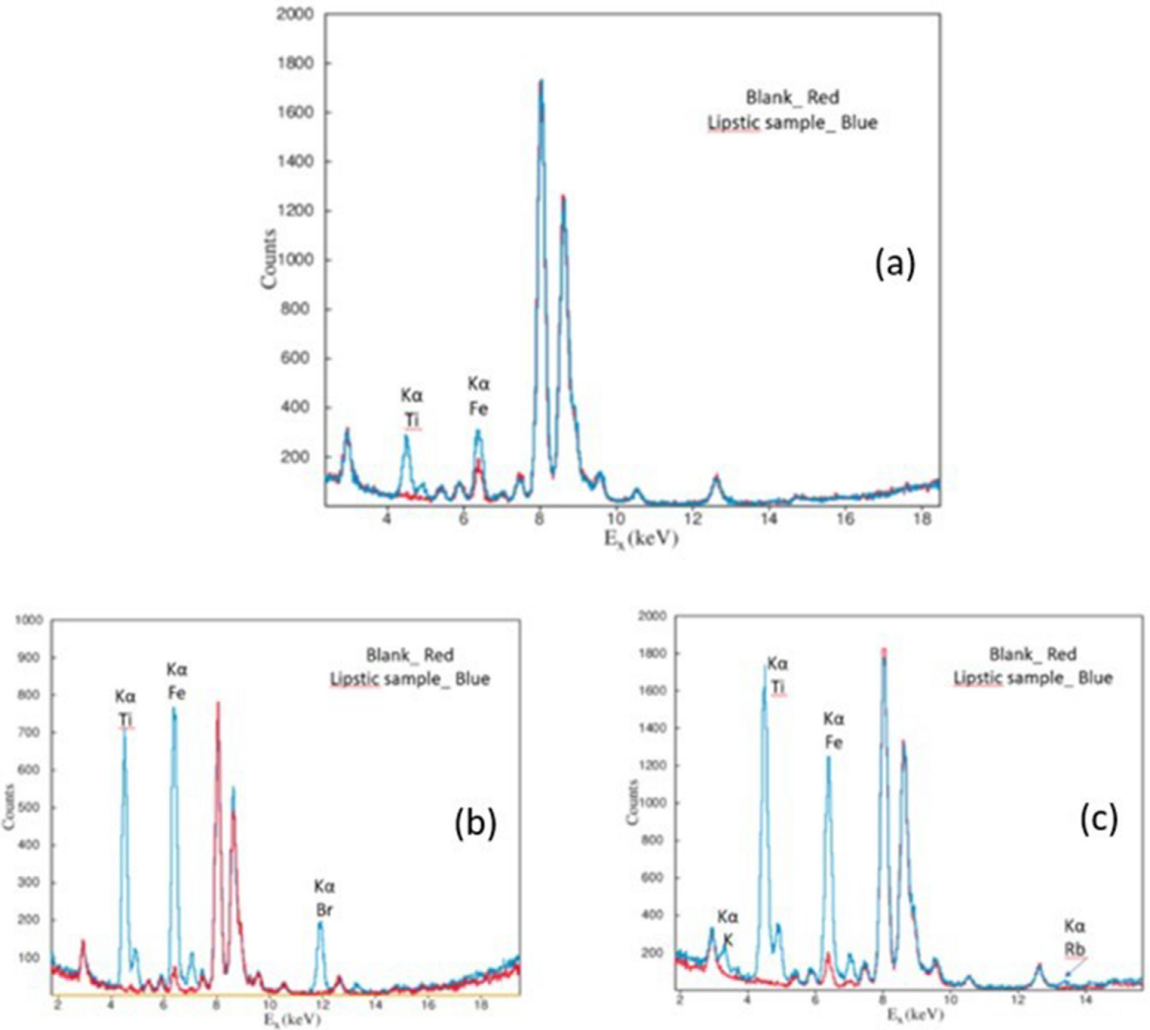
Characteristic spectra from three lipstick (thin target) samples. (a) S1, (b) S2, (c) S3. In each picture the red spectrum is from the Blank sample.

Three spectra (S1, S2 and S3) of different lipsticks in comparison with a blank sample are shown in “Fig. 7”.

**Fig. 8:**
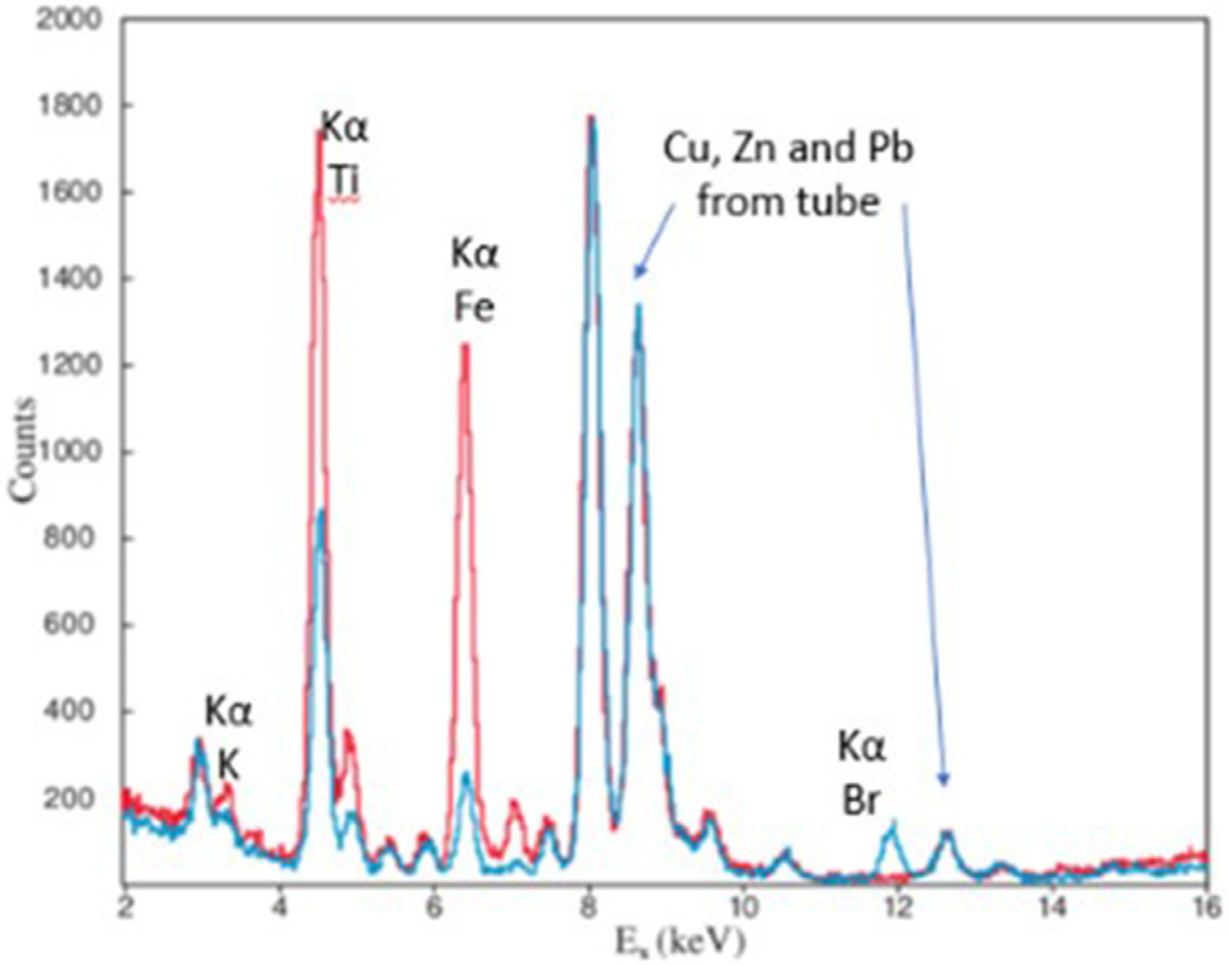
Overlapped spectra of two different lipsticks.

In all three samples, Fe and Ti are present. In sample S1 “Fig. 7(a)”, only Fe and Ti were detected. In sample S2 “Fig. 7(b)”, the characteristic peak of bromine (Kα 11.92 keV), is appeared additionally. In the third sample S3 potassium (K) and rubidium (Rb) were detected additionally to Fe and Ti. Other peaks present in the blank sample are due to argon (Ar) that exists in the air and the exciting X-ray tube (Fe, Cu, Zn, Pb). ND stands for not detected. In “Fig. 8”, two overlapped spectra of two different lipsticks under the same experimental conditions and approximately similar surface density are presented.

The presence of titanium in lipstics is due to TiO_2_ that it is used as physical UV filter and can be found in many personal care products. Furthermore, it is mostly used as pigment not only for personal care products but also in many other cases (paints, textiles, food, drugs and other) [17,18]. The presence of iron and bromine is due to their compounds that are used as colorants in many cosmetics [19]. Especially bromine compounds are commonly used in red and purple lipsticks and finally, rubidium is probably due to raw materials [15].

## Limitations

With our system, we may determine elements from Si and above. In practice the elements that could be detected depend on the detection limits. The detection limits depend on the signal-to-noise ratio, which can be altered by various parameters, such as the measurement time, the detector geometry, the intensity of the X-ray source, the matrix of the analyzed sample, etc. Here we provide indicative detection limits for the specific matrix and the specific conditions of measurements, for the elements that were detected by our system and also for Cu, Zn and Pb from which there is noise from the X-ray tube.

The Indicative detection limits (in ppm) were 600 for K, 150 for Ti and Cu, 100 for Fe and Zn, 10 for Br and Rb and 30 for Pb.

## Funding

The publication of the article in OA mode was financially supported in part by HEAL-Link and the Special Accounts for Research Grants of the University of West Attica.

## CRediT authorship contribution statement

**M Pilakouta:** Conceptualization, Methodology, Writing – original draft. **M Trapali:** Conceptualization, Methodology, Writing – original draft. **N Kallithrakas-Kontos:** Writing – review & editing.

## Declaration of competing interest

The authors declare that they have no known competing financial interests or personal relationships that could have appeared to influence the work reported in this paper.

## Data Availability

Data will be made available on request.

## Appendix Supplementary materials

Image, application 1

Image, application 2
