# Supplementary material for: XRF calibration with low-cost samples and implementation for quantification of inorganic elements in lipsticks
Source: MethodsX. 2024 Apr 7;12:102704. doi: 10.1016/j.mex.2024.102704 (PMC11033198; doi:10.1016/j.mex.2024.102704)

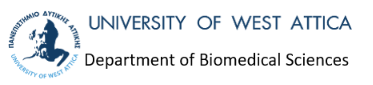

 **ChemBiochemCosm**

**XRF analysis of cosmetic products**

1. Preparation of Lipsticks, cremes and toothpaste samples

Samples of lipsticks, cremes and toothpastes can be analyzed without any other preparation, just applying a thin layer of each sample over a thin membrane. The samples should have a surface density less than 13 mg·cm^−2^ to be treated as “thin targets”.

Since the surface, Ac, of the cap we use is Ac=4.91 cm^2^, the net **DRY** sample mass should be less than ~65 mg.

- For lipsticks and toothpaste samples the starting mass should be about 10% higher. (~ 70 mg)
- Cremes losses about 70% of its weight when dry so the starting mass should be about 3.3 times higher (~ 200 mg)

1. Weight the cup with the membrane before applying the material. Wc=
2. Apply a thin layer of each sample homogenously on the membrane and weigth again
3. Find the net sample weight Wnet = W_CS_- W_C_ = and check if it is less than X* mg.
4. Left it to dry physically (24h), weight again, and find the net weight of the dry sample W_CSd_ =
5. Estimate the surface density of the sample. SD=

* depending on the sample

1. Data collection

Centre the sample on the opening of the XRF system.

With the educator help, select the irradiation conditions 40KV, 40μA and insert energy calibration in the DppMCA. For safety reasons, be sure that the distance between you and the XRF system is more than about half a meter.


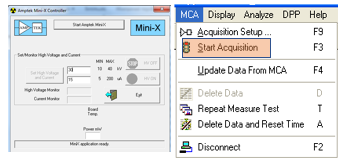


1. Start Acquisition. For Quick inspection select data ~500 or less sec. For quantification the yield of the peak of interest should be at least 500 counts.
2. Save the spectrum with an appropriate name.
3. Analysis

For a quick estimation use the DppMCA. For more accurate analysis ask educator to analyze with SPECTRW program.

Use the DppMCA program to find the centroid of the Kα photopeaks (Select (ROI) region of interest around the peaks and click on the maximum).


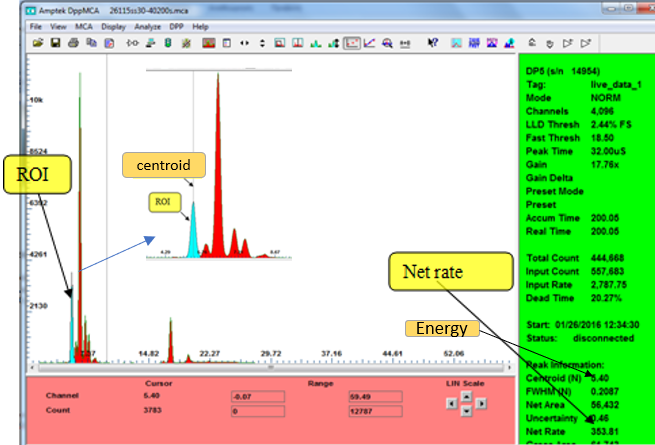


1. Record the Energy and the net rate results in the table
2. Use the X-ray Line Chart to identify peaks and qualitatively characterize the sample
3. Select the sensitivity factor for each detected element.
4. Find the elemental concentration of each element in the sample.
5. Compare the results of two lipsticks or cremes etc . Discuss the differences in the detected elements among samples.
6. Find in literature whether the detected elements are due to any of the ingredients of the examined product and how this substance affect its properties.

| **Sample:** | | | | | |
| --- | --- | --- | --- | --- | --- |
| Energy keV | Element | I (c/s) | Surface density of sample S_ds_  (mg/cm^2^) | Sensitivity factor of the element S_Fi_ | Ci=I* S_ds_/S_Fi_  ppm |
|  |  |  |  |  |  |
|  |  |  |  |  |  |
|  |  |  |  |  |  |
|  |  |  |  |  |  |
|  |  |  |  |  |  |

.


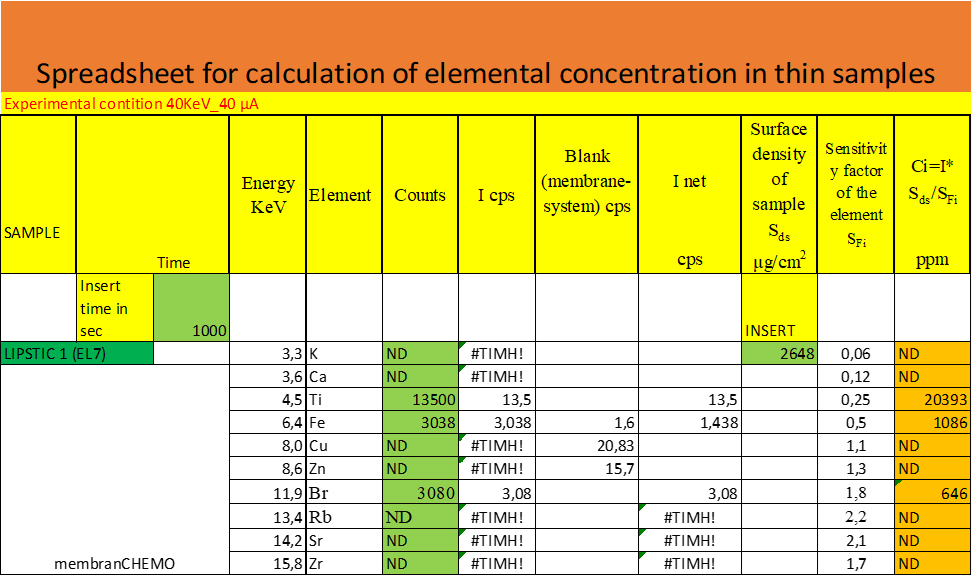


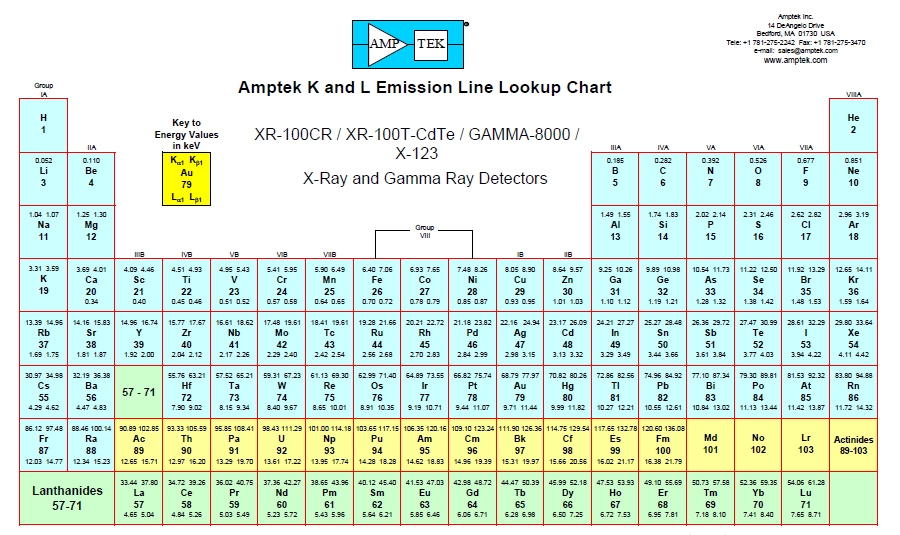

Supplement: Supplementary file 1 [file mmc1.docx]
