# Supplementary material for: XRF calibration with low-cost samples and implementation for quantification of inorganic elements in lipsticks
Source: MethodsX. 2024 Apr 7;12:102704. doi: 10.1016/j.mex.2024.102704 (PMC11033198; doi:10.1016/j.mex.2024.102704)
Supplement: Supplementary file 2 [file mmc2.docx]

UNIVERSITY OF WEST ATTICA

Department of Biomedical Sciences

Biochemistry Lab

*Preparation of stock solutions*

**Stock solutions from solid**

• First, we calculate the quantity (g) of the solid substance to create the requested

solution and weighing it on a balance.

• Add a small amount of deionized water to a volumetric flask of suitable volume.

• Dissolve the amount of solid substance in a small amount of deionized water in a beaker

boiling and transferring it to the volumetric flask. Successive washing of the dishes that

were used to dissolve the solid substance (beaker, funnel, rod).

• Add deionized water to the volumetric flask up to the mark using

water cannon and dropper.

• Stopper and shake the volumetric flask

**Stock solutions from a thicker solution**

• First, we calculate the quantity of the denser solution that will be used for the

creating the desired solution (dilution law).

• Add a small amount of deionized water to a volumetric flask of suitable volume.

• Using a transfer siphon and a safety pin, it can be transported calculated volume of the concentrated solution in the volumetric flask.

• Add deionized water to the volumetric flask up to the mark using water cannon and dropper.

• Stopper and shake the volumetric flask.

• Placing a label with the details of the solution (name, content, date preparation)


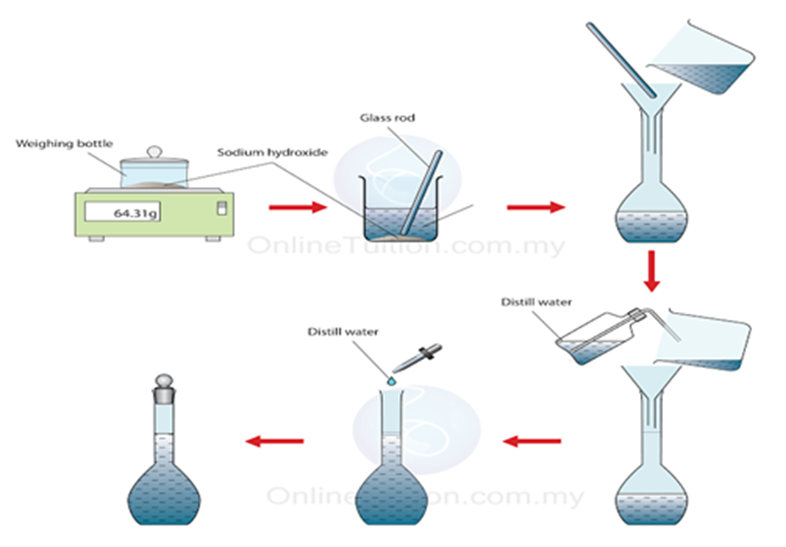


Experimental procedure for preparing a solution from a solid


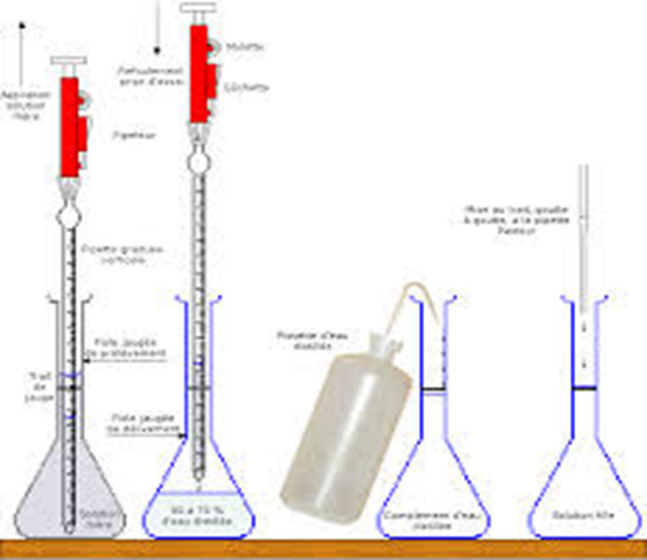


Experimental procedure for preparing a solution from a more concentrated solution (by dilution).

**Hazardous waste collection**

An important group of wastes are residues from chemical substances which are usually classified as hazardous waste. These substances are prohibited disposed of through municipal waste collection or the sewer system. The various types of waste categorized as hazardous waste are collected separately and delivered by the producer to approved companies. Hazardous waste is collected in special containers in compliance with the regulations (e.g. the Directive "Ordinance on the Hazardous Substances", reference also in the “Legal Conditions for the Handling of Hazardous Substances” and “Technical Guidelines on Safety in Chemical Laboratory Courses”. For each type of waste, they are used for collection of special containers supplied by the university. These containers are returned in the waste collection unit and the containers should be filled up to 90 % of capacity (to avoid the possibility of leakage during transport). The containers must be sealed and labeled appropriately. Companies are different collection point are not allowed to receive them. Also containers damaged, with leaks or externally contaminated with hazardous substances will not be accepted.
